# 6,6′-Dihydr­oxy-2,2′-[(propane-1,3-diyl­dioxy)bis­(nitrilo­methyl­idyne)]diphenol

**DOI:** 10.1107/S1600536808026731

**Published:** 2008-08-23

**Authors:** Wen-Kui Dong, Xue-Ni He, Yong-Hong Guan, Li Xu, Zong-Li Ren

**Affiliations:** a School of Chemical and Biological Engineering, Lanzhou Jiaotong University, Lanzhou 730070, People’s Republic of China

## Abstract

The mol­ecule of the title compound, C_17_H_18_N_2_O_6_, adopts a V-shaped conformation, the dihedral angle between the two halves of the mol­ecule being 81.31 (4) °. There is one half-mol­ecule in the asymmetric unit, with a crystallographic twofold rotation axis passing through the central C atom. There are strong intra­molecular O—H⋯N and O—H⋯O hydrogen bonds involving the hydr­oxy group and adjacent O and N atoms. In the crystal structure, inter­molecular O—H⋯O hydrogen bonds link the mol­ecules, forming an infinite three-dimensional supra­molecular structure.

## Related literature

For related literature, see: Akine *et al.* (2006 ▶); Dong & Feng (2006 ▶); Dong *et al.* (2008*a* ▶,*b* ▶,*c* ▶); Duan *et al.* (2007 ▶); Sharma (2002 ▶); Sun *et al.* (2004 ▶); Venkataramanan *et al.* (2005 ▶); Wang *et al.* (2007 ▶).
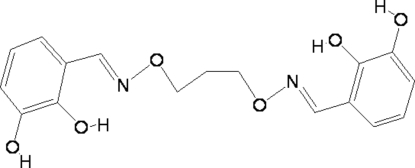


## Experimental

### 

#### Crystal data

C_17_H_18_N_2_O_6_
*M* *_r_* = 346.33
Monoclinic, 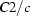

*a* = 27.836 (3) Å
*b* = 4.5949 (5) Å
*c* = 13.8081 (10) Å
β = 109.363 (2)°
*V* = 1666.2 (3) Å^3^
*Z* = 4
Mo *K*α radiation
μ = 0.11 mm^−1^
*T* = 298 (2) K
0.43 × 0.40 × 0.31 mm

#### Data collection

Brucker SMART 1000 CCD area-detector diffractometer
Absorption correction: multi-scan (*SADABS*; Sheldrick, 1996 ▶) *T*_min_ = 0.956, *T*_max_ = 0.968
4032 measured reflections
1476 independent reflections
1025 reflections with *I* > 2σ(*I*)
*R*_int_ = 0.030

#### Refinement

*R*[*F* ^2^ > 2σ(*F* ^2^)] = 0.066
*wR*(*F* ^2^) = 0.186
*S* = 1.05
1476 reflections
114 parameters
H-atom parameters constrained
Δρ_max_ = 0.20 e Å^−3^
Δρ_min_ = −0.25 e Å^−3^

### 

Data collection: *SMART* (Siemens, 1996 ▶); cell refinement: *SAINT* (Siemens, 1996 ▶); data reduction: *SAINT*; program(s) used to solve structure: *SHELXS97* (Sheldrick, 2008 ▶); program(s) used to refine structure: *SHELXL97* (Sheldrick, 2008 ▶); molecular graphics: *SHELXTL* (Sheldrick, 2008 ▶); software used to prepare material for publication: *SHELXTL*.

## Supplementary Material

Crystal structure: contains datablocks 2, I. DOI: 10.1107/S1600536808026731/pv2099sup1.cif

Structure factors: contains datablocks I. DOI: 10.1107/S1600536808026731/pv2099Isup2.hkl

Additional supplementary materials:  crystallographic information; 3D view; checkCIF report

## Figures and Tables

**Table 1:** Hydrogen-bond geometry (Å, °)

*D*—H⋯*A*	*D*—H	H⋯*A*	*D*⋯*A*	*D*—H⋯*A*
O2—H2⋯N1	0.82	1.94	2.650 (3)	144
O3—H3⋯O2	0.82	2.25	2.694 (4)	115
O3—H3⋯O1^i^	0.82	2.24	2.914 (4)	140

Symmetry code: (i) 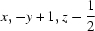.

**Fig. 1.**
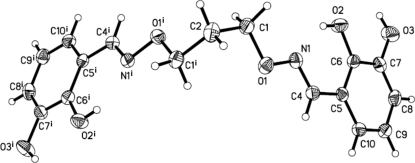
The molecular structure of the title compound with atom numbering scheme. Displacement ellipsoids for non-hydrogen atoms are drawn at the 30% probability level. [Symmetry code: -x + 1,y,-z + 5/2]

**Fig. 2.**
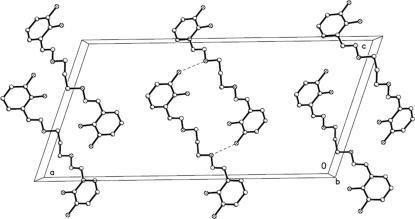
The packing diagram of the title compound showing intermolecular hydrogen bonds. H atoms are omitted for clarity.

## supplementary crystallographic information

### Comment

Salen-type compounds are chelate ligands, which have received great attention during the last decades (Sharma 2002; Akine *et al.*, 2006; Dong *et al.*, 2008*a*) due to their excellent complexing abilities towards various metal ions, especially in view of their potential use as ligands for preparation of functional complex materials (Venkataramanan *et al.*, 2005). They are widely used in supramolecular chemistry for the construction of some one-dimensional chains, two-dimensional planar or three-dimensional network structural supramolecular complexes. To our interest, some salen-type compounds can be used as elemental building blocks for construction of supramolecular structures *via* intermolecular hydrogen bonding or short contact interaction (Sun *et al.*, 2004; Akine *et al.*, 2006; Wang *et al.*, 2007). As an extension of our work (Dong & Feng 2006; Dong *et al.*, 2008*b*; Dong *et al.*, 2008*c*) on the structural characterization of salen-type bisoxime compounds, we report the structure of the title compound in this paper here.

The molecule of title compound adopts a V-shaped conformation with the dihedral angle between the two halves of the molecule is 81.31 (4) ° (Fig. 1). There is a half molecule in an asymmetric unit with a crystallographic twofold rotation axis passing through the central carbon of the three carbon atoms in the (—CH=N—O—(CH_2_)_3_—O—N=CH—) bridge. This structure is similar to what was observed in our previously reported salen-type bisoxime compound (Duan *et al.*, 2007). The dihedral angle formed by the two benzene rings in the molecule of the title compound is 82.22 (5) °. There are strong intramolecular O—H···N and O—H···O hydrogen bonds involving the hydroxy group and an adjacent O (or N) atoms (Table 1). In the crystal structure, intermolecular O—H···O hydrogen bonds link each molecule to 2 others into infinite three-dimensional supramolecular structure (Fig. 2), which is not similar to what was observed in our previously reported series salen-type compounds containing two- (Akine *et al.*, 2006) and five-methene (Dong *et al.*, 2008a) bridge.

### Experimental

6,6'-Dihydroxy-2,2'-[(pentane-1,5-diyldioxy)bis(nitrilomethylidyne)]diphenol was synthesized according to an analogous method reported earlier (Dong *et al.*, 2006; Dong *et al.*, 2008*a*). To an ethanol solution (5 ml) of 2,3-dihydroxybenzaldehyde (276.6 mg, 2.0 mmol) was added an ethanol solution (5 ml) of 1, 3-bis(aminooxy)propane (106.8 mg, 1.0 mmol). After the solution had been stirred at 328 K for 3 h, the mixture was filtered, washed successively with ethanol and ethanol/hexane (1:4), respectively. The product was dried under reduced pressure and purified by recrystallization from ethanol to yield 204.8 mg of pale-brown crystalline solid.

Pale-brown prismatical crystals of the title compound suitable for X-ray crystal analysis were grown up from a tetrahydrofuran-ethanol (3:4) mixed solution by slow evaporation of the solvent at room temperature.

### Refinement

Non-H atoms were refined anisotropically. H atoms were treated as riding atoms with distances C—H = 0.97 (CH_2_), or 0.93 Å (CH), O—H = 0.82 Å, and *U*_iso_(H) = 1.2 *U*_eq_(C) and 1.5 *U*_eq_(O).

### Crystal data

**Table:**

C_17_H_18_N_2_O_6_	*F*_000_ = 728
*M**_r_* = 346.33	*D*_x_ = 1.381 Mg m^−^^3^
Monoclinic, *C*2/*c*	Mo *K*α radiation λ = 0.71073 Å
Hall symbol: -C 2yc	Cell parameters from 1493 reflections
*a* = 27.836 (3) Å	θ = 2.8–27.7º
*b* = 4.5949 (5) Å	µ = 0.11 mm^−^^1^
*c* = 13.8081 (10) Å	*T* = 298 (2) K
β = 109.363 (2)º	Prismatic, pale-brown
*V* = 1666.2 (3) Å^3^	0.43 × 0.40 × 0.31 mm
*Z* = 4	

### Data collection

**Table:**

Brucker SMART 1000 CCD area-detector diffractometer	1476 independent reflections
Radiation source: fine-focus sealed tube	1025 reflections with *I* > 2σ(*I*)
Monochromator: graphite	*R*_int_ = 0.030
*T* = 298(2) K	θ_max_ = 25.0º
phi and ω scans	θ_min_ = 1.6º
Absorption correction: multi-scan(SADABS; Sheldrick, 1996)	*h* = −32→27
*T*_min_ = 0.956, *T*_max_ = 0.968	*k* = −5→5
4032 measured reflections	*l* = −16→16

### Refinement

**Table:**

Refinement on *F*^2^	Secondary atom site location: difference Fourier map
Least-squares matrix: full	Hydrogen site location: inferred from neighbouring sites
*R*[*F*^2^ > 2σ(*F*^2^)] = 0.066	H-atom parameters constrained
*wR*(*F*^2^) = 0.186	*w* = 1/[σ^2^(*F*_o_^2^) + (0.0856*P*)^2^ + 2.8739*P*] where *P* = (*F*_o_^2^ + 2*F*_c_^2^)/3
*S* = 1.05	(Δ/σ)_max_ < 0.001
1476 reflections	Δρ_max_ = 0.20 e Å^−^^3^
114 parameters	Δρ_min_ = −0.25 e Å^−^^3^
Primary atom site location: structure-invariant direct methods	Extinction correction: none

### Special details

**Table:**

Experimental. Yield, 59.1%, mp. 425–427 K. Anal. Calc. for C_17_H_18_N_2_O_6_: C, 59.96; H, 5.24; N, 8.09. Found: C, 60.17; H, 5.31; N, 7.92.
Geometry. All e.s.d.'s (except the e.s.d. in the dihedral angle between two l.s. planes) are estimated using the full covariance matrix. The cell e.s.d.'s are taken into account individually in the estimation of e.s.d.'s in distances, angles and torsion angles; correlations between e.s.d.'s in cell parameters are only used when they are defined by crystal symmetry. An approximate (isotropic) treatment of cell e.s.d.'s is used for estimating e.s.d.'s involving l.s. planes.
Refinement. Refinement of *F*^2^ against ALL reflections. The weighted *R*-factor *wR* and goodness of fit *S* are based on *F*^2^, conventional *R*-factors *R* are based on *F*, with *F* set to zero for negative *F*^2^. The threshold expression of *F*^2^ > σ(*F*^2^) is used only for calculating *R*-factors(gt) *etc*. and is not relevant to the choice of reflections for refinement. *R*-factors based on *F*^2^ are statistically about twice as large as those based on *F*, and *R*- factors based on ALL data will be even larger.

### Fractional atomic coordinates and isotropic or equivalent isotropic displacement parameters (Å^2^)

**Table:**

	*x*	*y*	*z*	*U*_iso_*/*U*_eq_	Occ. (<1)
O1	0.43592 (8)	0.2306 (5)	1.15521 (16)	0.0466 (6)	
O2	0.41085 (8)	0.6412 (6)	0.88832 (17)	0.0539 (7)	
H2	0.4236	0.5367	0.9385	0.081*	
O3	0.36020 (10)	1.0086 (6)	0.73677 (18)	0.0671 (9)	
H3	0.3855	0.9080	0.7439	0.101*	
N1	0.41732 (9)	0.4035 (6)	1.06706 (18)	0.0413 (7)	
C1	0.48064 (12)	0.0823 (7)	1.1531 (2)	0.0456 (8)	
H1A	0.5063	0.2214	1.1500	0.055*	
H1B	0.4727	−0.0431	1.0933	0.055*	
C2	0.5000	−0.0961 (11)	1.2500	0.0529 (13)	
H2A	0.4727	−0.2207	1.2545	0.063*	0.50
H2B	0.5273	−0.2207	1.2455	0.063*	0.50
C4	0.37794 (11)	0.5439 (7)	1.0649 (2)	0.0409 (8)	
H4	0.3650	0.5210	1.1185	0.049*	
C5	0.35228 (11)	0.7400 (7)	0.9813 (2)	0.0372 (7)	
C6	0.36954 (11)	0.7802 (7)	0.8979 (2)	0.0387 (8)	
C7	0.34391 (12)	0.9687 (7)	0.8197 (2)	0.0446 (8)	
C8	0.30183 (13)	1.1161 (8)	0.8225 (3)	0.0499 (9)	
H8	0.2850	1.2423	0.7695	0.060*	
C9	0.28425 (12)	1.0774 (8)	0.9046 (3)	0.0490 (9)	
H9	0.2555	1.1772	0.9066	0.059*	
C10	0.30912 (12)	0.8929 (8)	0.9822 (2)	0.0443 (8)	
H10	0.2971	0.8682	1.0369	0.053*	

### Atomic displacement parameters (Å^2^)

**Table:**

	*U*^11^	*U*^22^	*U*^33^	*U*^12^	*U*^13^	*U*^23^
O1	0.0425 (12)	0.0521 (15)	0.0435 (13)	0.0042 (11)	0.0119 (10)	0.0151 (11)
O2	0.0523 (14)	0.0655 (16)	0.0510 (14)	0.0138 (12)	0.0267 (11)	0.0107 (12)
O3	0.0821 (19)	0.0788 (19)	0.0470 (15)	0.0177 (15)	0.0303 (13)	0.0176 (13)
N1	0.0411 (15)	0.0424 (16)	0.0377 (14)	−0.0041 (13)	0.0096 (11)	0.0029 (12)
C1	0.0406 (18)	0.044 (2)	0.0480 (19)	0.0026 (15)	0.0094 (15)	−0.0031 (16)
C2	0.049 (3)	0.044 (3)	0.056 (3)	0.000	0.005 (2)	0.000
C4	0.0387 (17)	0.047 (2)	0.0377 (17)	−0.0033 (15)	0.0133 (14)	−0.0002 (15)
C5	0.0364 (16)	0.0367 (18)	0.0360 (16)	−0.0084 (14)	0.0088 (13)	−0.0060 (14)
C6	0.0352 (16)	0.0391 (18)	0.0407 (17)	−0.0009 (14)	0.0110 (13)	−0.0033 (15)
C7	0.051 (2)	0.047 (2)	0.0352 (17)	−0.0083 (16)	0.0123 (15)	−0.0008 (15)
C8	0.0468 (19)	0.048 (2)	0.0446 (19)	0.0064 (17)	0.0017 (15)	0.0040 (16)
C9	0.0396 (18)	0.054 (2)	0.050 (2)	0.0042 (17)	0.0106 (15)	−0.0062 (18)
C10	0.0418 (18)	0.050 (2)	0.0429 (18)	−0.0044 (16)	0.0165 (14)	−0.0055 (16)

### Geometric parameters (Å, °)

**Table:**

O1—N1	1.401 (3)	C2—H2B	0.9700
O1—C1	1.428 (4)	C4—C5	1.452 (4)
O2—C6	1.359 (4)	C4—H4	0.9300
O2—H2	0.8207	C5—C10	1.395 (4)
O3—C7	1.377 (4)	C5—C6	1.400 (4)
O3—H3	0.8195	C6—C7	1.383 (4)
N1—C4	1.264 (4)	C7—C8	1.365 (5)
C1—C2	1.508 (4)	C8—C9	1.387 (5)
C1—H1A	0.9700	C8—H8	0.9300
C1—H1B	0.9700	C9—C10	1.362 (5)
C2—C1^i^	1.508 (4)	C9—H9	0.9300
C2—H2A	0.9700	C10—H10	0.9300
			
N1—O1—C1	109.0 (2)	C10—C5—C6	118.2 (3)
C6—O2—H2	109.7	C10—C5—C4	120.2 (3)
C7—O3—H3	109.3	C6—C5—C4	121.6 (3)
C4—N1—O1	112.2 (2)	O2—C6—C7	116.9 (3)
O1—C1—C2	107.4 (2)	O2—C6—C5	123.4 (3)
O1—C1—H1A	110.2	C7—C6—C5	119.7 (3)
C2—C1—H1A	110.2	C8—C7—O3	118.7 (3)
O1—C1—H1B	110.2	C8—C7—C6	121.0 (3)
C2—C1—H1B	110.2	O3—C7—C6	120.3 (3)
H1A—C1—H1B	108.5	C7—C8—C9	119.9 (3)
C1^i^—C2—C1	114.2 (4)	C7—C8—H8	120.1
C1^i^—C2—H2A	108.7	C9—C8—H8	120.1
C1—C2—H2A	108.7	C10—C9—C8	119.9 (3)
C1^i^—C2—H2B	108.7	C10—C9—H9	120.1
C1—C2—H2B	108.7	C8—C9—H9	120.1
H2A—C2—H2B	107.6	C9—C10—C5	121.4 (3)
N1—C4—C5	122.0 (3)	C9—C10—H10	119.3
N1—C4—H4	119.0	C5—C10—H10	119.3
C5—C4—H4	119.0		
			
C1—O1—N1—C4	−179.2 (3)	O2—C6—C7—C8	179.6 (3)
N1—O1—C1—C2	178.9 (3)	C5—C6—C7—C8	−0.2 (5)
O1—C1—C2—C1^i^	−66.8 (2)	O2—C6—C7—O3	0.3 (5)
O1—N1—C4—C5	179.3 (3)	C5—C6—C7—O3	−179.5 (3)
N1—C4—C5—C10	−179.5 (3)	O3—C7—C8—C9	179.3 (3)
N1—C4—C5—C6	0.9 (5)	C6—C7—C8—C9	0.0 (5)
C10—C5—C6—O2	−179.4 (3)	C7—C8—C9—C10	0.1 (5)
C4—C5—C6—O2	0.2 (5)	C8—C9—C10—C5	0.0 (5)
C10—C5—C6—C7	0.3 (4)	C6—C5—C10—C9	−0.2 (5)
C4—C5—C6—C7	179.9 (3)	C4—C5—C10—C9	−179.8 (3)

Symmetry codes: (i) −*x*+1, *y*, −*z*+5/2.

### Hydrogen-bond geometry (Å, °)

**Table:**

*D*—H···*A*	*D*—H	H···*A*	*D*···*A*	*D*—H···*A*
O2—H2···N1	0.82	1.94	2.650 (3)	144
O3—H3···O2	0.82	2.25	2.694 (4)	115
O3—H3···O1^ii^	0.82	2.24	2.914 (4)	140

Symmetry codes: (ii) *x*, −*y*+1, *z*−1/2.
